# Smoking and Cancer in Poland

**DOI:** 10.1038/bjc.1960.46

**Published:** 1960-09

**Authors:** J. Staszewski


### SMOKING AND CANCER IN POLAND

J. STASZEWSKI

*From the Institute of Oncology, ul. Czerwonej Armii 15, Gliwice, Poland*

### PART I.—STATISTICAL DATA ON SMOKING AND "TOBACCO TRACT" CANCER IN POLAND

Received for publication March 7, 1960

THE aim of this study is to discuss the results of the investigations on the connection between smoking and the appearance of some cancers. The investigations were carried out on patients of the Institute of Oncology in Gliwice which is the largest centre treating tumours in Poland.

In the first part of the study a short presentation is given of the consumption of tobacco in Poland and the appearance of " tobacco tract " cancer.

The view that smoking is a frequent cause of the appearance of lung cancer and some other tumours is ever increasing; it is not however accepted by all. That is why exhaustive investigations should be continued on the connection between smoking and the appearance of cancer.

If a distinct connection between smoking and for instance lung cancer is always found during the investigations in a variety of environments and in different countries then this fact cannot be considered as accidental. Such investigations should also make possible the recognition of the degree of the association between smoking and cancer morbidity (chances of morbidity depending upon the smoking habits) and explain what percentage of cases is in the given environment connected with smoking. If this percentage is greater in the environments with higher consumption of tobacco and lower in the environments in which act other carcinogenic factors—this will be the second serious argument for the carcinogenic action of tobacco smoke.

The third argument would be the finding of a connection between smoking and epidermoid cancer of the entire "tobacco tract", of the whole mucous membrane immediately in touch with tobacco smoke. It is difficult to imagine that such a connection could be accidental. Interesting results should be obtained by finding out the degree of this connection for cancer of various localizations and by comparison of the data obtained with the localization of tobacco tar in the respiratory tract of smokers (such studies have been started by Ermala and Holsti (1955).

If, as some claim, the connection between smoking and "tobacco tract" cancer is not a causative connection then it would have to be the effect of interdependence between smoking and some unknown factor causing the appearance of these tumours. The investigation of the connection between smoking and cancer should enable one to detect such a factor.

### A. *Consumption of tobacco in Poland*

Some data concerning the consumption of tobacco in Poland are presented in Tables I and II. They show a marked increase in the consumption of tobacco (mostly cigarettes) after the second World War.

TABLE I.—*Tobacco Consumption in Poland (Polish and Imported Tobacco)—According to the Data Obtained from the Polish Tobacco Industry*

<table>

<tr><td>Total consumption per head of the population (grammes of tobacco products)</td><td>603</td><td>692</td><td>549</td><td>562</td><td>849</td><td>1,216</td><td>1,491</td></tr>

</table>

*Note:* In order to make the table smaller the data are presented for every fourth year. For 1938–44 there are no data available; for 1945–47 the data are not accurate and for that reason are not presented here.

TABLE II.—*Tobacco Consumption in Poland in the Part Previously under Russian Rule (Doleżal, 1922)*

| Year | Number of inhabitants | Tobacco consumption (pounds per head) |
|---|---|---|
| 1829 | 4,137,634 | 0·72 |
| 1831 | 3,762,003 | 0·73 |
| 1832 | 3,914,665 | 0·94 |
| 1833 | 4,037,952 | 1·01 |
| 1834 | 4,059,517 | 0·83 |
| 1835 | 4,188,112 | 0·70 |
| 1845 | 4,798,658 | 0·79 |
| 1851 | 4,810,735 | 0·80 |
| 1855 | 4,647,454 | 0·93 |
| 1859 | 4,747,454 | 0·86 |

During the period between the first and the second World War mainly hand-made cigarettes were smoked (in cigarette tubes or rolled in tissue paper). Since the second World War hand-made cigarettes have been almost entirely given up for factory-made cigarettes. The smoking of pipes and cigars, disappearing at present, was rather popular during the period between the wars in the western part of Poland (Pomerania, Poznań District, Silesia, i.e. in the previous Prussian occupation[1]) but rarely met in the remaining parts of Poland.

Until recently there were no data on smoking habits of the individual smoker or on the structure of tobacco consumption in Poland. It is estimated that before the second World War the smokers constituted about 20 per cent of the entire population and that after the war the percentage increased mainly by a greater number of women smokers (personal communication from the Polish Tobacco Industry).

In 1957 preliminary investigations were carried out concerning the smoking habits and the structure of tobacco consumption in Poland (Staszewski and Wiśniewski, 1960). During these investigations the author collected data both on smoking habits (beginning, breaks, intensity and the manner of smoking)

[1] Up to 1918 the territory of Poland was devided for over 100 years by three occupants: Prussia, Russia and Austria.

as well as on such background data as professional activities, the places of residence (past and present) and the diseases the smokers had in the past.

Thus 2725 individuals of over 20 years of age were examined whose calling at the Institute of Oncology in Gliwice was most probably not connected with smoking as a causative agent: 1813 women (cancer and other diseases of the mammae—620, genital organs—600, skin—410, other organs which were not exposed to the direct action of the tobacco smoke—183) and 912 men (skin cancer—405, other skin diseases—117, cancer and other diseases of the genitals and mammary glands—82, lymphatic system—80, large intestine and rectum—71, skeletal system and soft tissues—53, other organs—104).

82·7 per cent of the examined men were smokers (present or past) who usually started smoking at about 20; the percentage of the smokers increased with age up to 60–70 years of life, the average intensity of smoking on the other hand decreased with age. Among women there were 8·4 per cent smokers. They had started smoking later and at various ages (average about 26 years old); the percentage of the smokers increased up to 35 years of age and then distinctly decreased. Differences were found in the consumption of tobacco between the different professional groups in both sexes. No distinct differences in smoking habits were noted between men living in the cities and in rural areas, but among women living in the rural areas there were markedly fewer smokers (1·8 per cent) than among those living in cities (12·8 per cent). Pipe and cigar smoking was met mainly among the population of Silesia (particularly among coal-miners and foundry workers and markedly fewer pipe smokers among office workers); the frequency of pipe smoking increased with age; it was not met before the thirty-fifth year of life, nor among women. 91·5 per cent of smokers who smoked cigarettes exclusively inhaled smoke as against only 34·7 per cent of those who smoked only pipe and/or cigars and 68·3 per cent of mixed smokers.

Some other data obtained will be presented by the Tables of the second part of this study.

B. *" Tobacco tract " cancer in Poland*

Up to 1950 the nomenclature of diseases and causes of death in Poland classified tumours only into 2 groups: " carcinoma and other malignant tumours ", and " benign tumours or not diagnosed as malignant ". Starting with 1951 there is a more exact classification taking into account, among others, the group of " carcinoma and other malignant tumours of the organs of respiration ". Data on the mortality caused by the tumours of this group are shown in Table III. We can see a distinct increase both in the number of deaths due to cancer of the organs of respiration as well as an increase in their percentage in comparison with the total number of deaths or with the deaths due to all tumours. There is also a constant and distinct increase in the preponderance of men.

Since lung cancer, constituting the largest number of patients in this group, is curable so far in only a small percentage of the cases and the survival after the diagnosis amounts on the average to several months, it can be accepted that mortality in this case is only slightly smaller than morbidity.

Large differences in the mortality (and thus in morbidity) between the inhabitants of the capital or industrial Silesia (Katowice District) and agricultural districts (as Kielce District lying more or less half-way between Warsaw and Silesia) are most probably brought about first of all by the differences in the

TABLE III.—*Respiratory Cancer Mortality (No. V—99 Nomenclature of Diseases and Causes of Death), According to the Data Obtained from the Central Office of Statistics*

| Year | Number of deaths due to cancer of the respiratory organs: Total | Men | Women | Percentage of deaths due to respiratory cancer — In comparison with total mortality: Poland | City of Warsaw | Katowice District | Kielce District | In comparsion with mortality due to tumours: Poland | City of Warsaw | Katowice District | Kielce District |
|---|---|---|---|---|---|---|---|---|---|---|---|
| 1951 | 944 | 663 | 281 | 0·30 | 1·23 | 0·54 | 0·09 | 6·01 | 10·01 | 7·37 | 3·19 |
| 1952 | 1035 | 746 | 289 | 0·36 | 1·09 | 0·68 | 0·15 | 6·45 | 8·67 | 7·98 | 4·72 |
| 1953 | 1278 | 924 | 354 | 0·48 | 1·58 | 0·73 | 0·28 | 7·58 | 10·50 | 8·26 | 7·71 |
| 1954 | 1472 | 1113 | 359 | 0·53 | 1·75 | 0·80 | 0·20 | 8·19 | 12·17 | 8·93 | 5·19 |
| 1955 | 1577 | 1190 | 387 | 0·60 | 1·70 | 0·89 | 0·25 | 8·16 | 10·12 | 8·82 | 5·42 |
| 1956 | 1828 | 1418 | 410 | 0·73 | 2·23 | 1·24 | 0·32 | 8·96 | 12·34 | 10·87 | 6·32 |
| 1957 | 2041 | 1530 | 511 | 0·76 | 2·37 | 1·16 | 0·39 | 9·30 | 14·11 | 10·54 | 7·70 |

detection of tumours—difficult from the diagnostic point of view (as lung cancer). The data presented for the whole territory of Poland should therefore be considered as lower than in reality and the mortality and morbidity rates for Warsaw or Silesia are probably the closest to the actual rates for the whole country.

Since 1954 there exists in Poland a regulation requiring the notification of new cases of malignant tumours. It is not however strictly observed, e.g. in 1956 there were in Poland 20,409 deaths due to tumours (according to death statistics) but only 17,796 newly detected cases of malignant tumours were registered.

Lung cancer was, in 1956, the fourth in frequency of registered newly detected cases of malignant tumours among males (617 cases), being after cancer of the stomach (1444 cases), skin (822) and lip (747) and before cancer of the intestines (296 cases), larynx (270), oesophagus (220) and other organs.

The following are some data taken from the post-mortem statistics:

Nowicki (1931) claims that during the years 1896–1930 out of 30,957 post-mortem examinations performed at the Institute of Pathological Anatomy of the Jan Kazimierz University in Lwów, the percentage of lung cancer (93 cases) increased from 0·07 to 0·47. Out of a total of 2047 tumours the percentage of lung cancer increased from 1·8 to 5·9. Syrek (1931) mentions that during 1901–30 in the Department of Pathological Anatomy of the Jagiellonian University in Cracow, out of 28,455 post-mortem examinations 86 cases were found to be lung cancer (67 men and 19 women). The proportion of appearance of this tumour to the total number of post-mortem investigations increased during this period from 0·09 to 0·60 per cent, and in reference to the total number of cancer from 1·36 to 7·18 per cent.

Dąbrowska, Połtorzycka and Trojanowska (1932) presented data from 3 Warsaw Departments in which in the years 1911–31 out of 25,639 post-mortems performed in 212 cases lung cancer was found (169 men and 43 women). The frequency of lung cancer in respect to the total number of post-mortems increased from 0·5 per cent during the years 1911–13 to 1·4 per cent during 1929–31 and in respect to post-mortems due to malignant tumours from 5·7 to 12·8 per cent.

Thus one can see that the increase in the frequency of appearance of lung cancer is apparent also on the ground of post-mortem statistics.

*Summary*

The importance of epidemiological investigations into the problem of the carcinogenic action of tobacco smoking is presented.

Certain data are given in reference to tobacco consumption and smoking habits, concerning statistics on " tobacco tract " cancer mortality, and on post-mortem statistics of lung cancer in Poland.

### REFERENCES

DĄBROWSKA, J., POŁTORZYCKA, S. AND TROJANOWSKA, A.—(1932) *Gruźlica*, **7**, 259.
DOLEŻAL, F.—(1922) in ' Leopold Kronberg '. Warszawa.
ERMALA, P. AND HOLSTI, L. R.—(1955) *Cancer*, **8**, 673.
NOWICKI, W.—(1931) *Polsk. Gaz. lek.*, **10**, 937.
STASZEWSKI, J. AND WIŚNIEWSKI, K.—(1960) *Roczn. Nauk rol.*, in press.
SYREK, A.—(1931) *Polsk. Gaz. lek.*, **10**, 995.

### PART II.—TOBACCO SMOKING AND LUNG CANCER

### *Materials, Methods, Definitions*

In more than 4 years between July 1954 and October 1958, 1031 men and 143 women were admitted to the Institute of Oncology in Gliwice with the diagnosis or suspicion of lung cancer.

I interviewed these patients as to the smoking habits, professions and places of residence in the same manner as the control group discussed in Part I of this study.

In 298 patients (275 men and 23 women) diagnosis of lung cancer was confirmed by histopathological findings. Seventy-one patients (including 15 men and 3 women with histopathologically proved lung cancer) were not interviewed because of their too short stay at the Institute. There remain then 281 patients (260 men and 21 women) interviewed with diagnosis confirmed histopathologically. These patients are the subject of the present study. Our material comprises over a half of the cases registered in Poland and possessing histopathological confirmation, e.g. in 1956 there were 127 such cases including 68 cases from our material.

The patients were divided into groups according to the histopathological diagnosis: 1. squamous-cell carcinoma, 2. adenocarcinoma, 3. microcellular carcinoma, 4. undifferentiated carcinoma or a type histologically not determined (foci carcinomatosi). The general characteristics of men belonging to these groups as well as to the control group are presented in Table I.

Table II presents the smoking habits of men suffering from lung cancer, and also the evaluation of statistical significance of the differences between them and the numbers characterizing smoking habits of the control group.

Those individuals who smoked for at least a year—and not less than an average of 1 g. of tobacco a day—were considered as " smokers ". The individuals who besides cigarettes smoked pipe and/or cigars (each one smoked in a sufficient amount to consider the individual as a " smoker ") were called " mixed smokers ".

TABLE I.—*General Characteristics of Men Suffering from Lung Cancer and of the Control Group*

| | Squamous cell carcinoma | Adeno-carcinoma | Micro-cellular carcinoma | Undiffer-entiated carcinoma or histological type not settled | Total carcinoma of bronchus | Control group |
|---|---|---|---|---|---|---|
| Number of individuals examined | 137 | 20 | 32 | 71 | 260 | 912 |
| Average age . . . . . | 56·5 | 52·8 | 50·7 | 55·6 | 55·3 | 53·4 |
| Number and % of inhabitants of towns | 85 (62·0) | 12 (60·0) | 24 (75·0) | 46 (64·8) | 167 (64·2) | 568 (62·3) |
| Number and % of inhabitants of Upper Silesia | 104 (75·9) | 15 (75·0) | 24 (75·0) | 49 (69·0) | 192 (73·8) | 641 (70·3) |
| Number and % of office workers | 29 (21·2) | 3 (15·0) | 7 (21·9) | 16 (22·5) | 55 (21·2) | 111 (12·2) |
| Number and % of farmers . | 7 (5·1) | 4 (20·0) | 4 (12·5) | 7 (9·9) | 22 (8·5) | 126 (13·8) |

TABLE II.—*Smoking Habits of Men Suffering from Lung Cancer and of the Control Group*

| | Squamous cell carcinoma | Adeno-carcinoma§ | Micro-cellular carcinoma§ | Undiffer-entiated carcinoma or histclogical type not settled | Total carcinoma of bronchus | Control group |
|---|---|---|---|---|---|---|
| Number of individuals examined | 137 | 20 | 32 | 71 | 260 | 912 |
| Number and % of smokers . | 137 (100·0)‡ | 20 (100·0) | 29 (90·6) | 69 (97·2)‡ | 255 (98·1)*‡ | 754 (82·7) |
| Average intensity of smoking . | 17·2‡ | 16·0 | 15·0 | 15·9† | 16·5*‡ | 12·2 |
| Average index of smoking . | 666·2‡ | 510·5 | 493·4 | 587·6† | 612·0‡ | 406·3 |
| Number and % of heavy smokers (with the index over 300) | 133 (97·1)‡ | 17 (85·0) | 24 (82·8) | 61 (88·4)‡ | 235 (92·2)‡ | 447 (59·3) |
| Number and % cf smokers smoking only cigarettes . | 117 (85·4)† | 15 (75·0) | 23 (79·3) | 63 (91·3)† | 218 (85·5)† | 552 (73·2) |
| Number and % of smokers smoking only pipe and/or cigars | 2 (1·45)‡ | 0 (0·0) | 2 (6·9) | 0 (0·0) | 4 (1·6)‡ | 101 (13·4) |
| Number and % of smokers inhaling smoke | 133 (97·1)‡ | 18 (90·0) | 28 (96·6) | 66 (95·6) † | 245 (96·1)‡ | 609 (80·8) |
| Average duration of smoking habits§ | 38·7 | 32·9 | 31·0 | 38·1 | 37·1 | 32·2 |

* The calculations of significance have been taken from the study by Dr. Różanowicz (unpublished).

† The difference with the control group is significant—exceeding over three times the standard error of difference calculated in the equation :

$$SE = \sqrt{\frac{p_1 \cdot q_1}{n_1} + \frac{p_2 \cdot q_2}{n_2}}$$

‡ The difference with the control group is exceedingly significant, exceeding at least six times the standard error of difference.

§ For adenocarcinoma, for microcellular cancer, and for average duration of smoking habits, significance of differences was not computed.

The duration of smoking was determined after subtracting the breaks in smoking lasting longer than a year.

The intensity of smoking or the amount of grammes of tobacco smoked daily is given as an average for the whole period of smoking. It was accepted that 1 cigarette = 1 g. of tobacco, and 1 cigar = 4 g. of tobacco.

The most frequently met classification of the individuals in reference to smoking habits is the one according to the intensity of smoking (e.g. smoking 1 package of cigarettes, or smoking more than 1 package a day, etc.). This does not however take into account a very important factor of time, and causes placing together into one group smokers who all smoke more than 1 package of cigarettes a day indeed—but one of them smokes for 5 years while another one for 40 years. In order to avoid this and for the purpose of better characterization of smoking by one number—giving some idea as to the intensity and the duration of the smoking habit as well—the term " smoking index " was introduced (Staszewski, 1958). This index is the product of intensity of smoking multiplied by the duration of smoking. If the individual smoked, for example, 12 cigarettes a day on the average for 40 years then the smoking index would be $12 \times 40 = 480$. This index multiplied by 0·365 gives the amount of tobacco in kilograms smoked during the life of the individual.

Those who presented the smoking index over 300 were considered as " heavy smokers ".

Individuals living steadily in a town or usually in a town and in the country not longer than 10 years, are considered as " town inhabitants ".

Individuals who lived steadily in Katowice or Opole Districts or not longer than 10 years outside of these Districts are considered as " inhabitants of Upper Silesia ".

For the classification of the individuals according to occupations, the occupation performed the longest was taken into consideration.

### *Results*

A. *Men* (Table II)

The percentage of smokers was considerably higher among the individuals afflicted by lung cancer than in the control group. It is striking that there is not one non-smoker among patients with squamous-cell carcinoma, who constitute over 50 per cent of patients with lung cancer.

The average smoking index among smokers was 50 per cent higher in patients with lung cancer than in the control group.

The percentage of " heavy " smokers (i.e. with the smoking index over 300) was also about 50 per cent higher in patients with lung cancer.

Average intensity of smoking among patients with lung cancer was about one-third higher than in the control group.

The average duration of smoking depends on three factors: Average age of the persons examined (among patients with lung cancer it was almost 2 years higher than in the control group), breaks in smoking, and the age of beginning smoking.

Individuals with lung cancer began smoking on the average 1·8 years earlier than those in the control group.

TABLE III.—*Smoking Habits of Women Suffering from Lung Cancer and of the Control Group*

| | Squamous cell carcinoma | Adeno-carcinoma | Micro-cellular carcinoma | Undifferentiated carcinoma or histological type not settled | Total carcinoma of bronchus | Control group |
|---|---|---|---|---|---|---|
| Number of individuals examined | 1 | 11 | 5 | 4 | 21 | 1813 |
| Number and % of smokers . | 1 (100·0) | 1 (9·1) | 1 (20·0) | 3 (75·0) | 6 (28·6) | 153 (8·4) |
| Average index of smoking (calculated for smokers) | 540 | 30 | 390 | 371·7 | 345·8 | 142·6 |
| Average index of smoking (calculated for smokers and non-smokers together) | 540 | 2·7 | 78·0 | 278·8 | 98·8 | 12·0 |
| Number and % of heavy smokers (with the index over 300) | 1 (100·0) | 0 (0·0) | 1 (100·0) | 2 (66·7) | 4 (66·7) | 20 (13·2) |
| % of individuals examined (smokers and non-smokers together) being heavy smokers | 100·0 | 0·0 | 20·0 | 50·0 | 19·0 | 1·2 |
| Average intensity of smoking . | 15 | 3 | 10 | 15·7 | 12·5 | 8·5 |

Among 137 patients with squamous-cell carcinoma only 2 smoked less than 25 years: one—14 years (a year after pneumonectomy performed in the thirty-third year of life carcinoma clarocellulare renis was found); the second—21 years. Among the patients with adenocarcinoma 5 patients had smoked for less than 25 years, among the patients with microcellular carcinoma—3, with undifferentiated—4.

*The manner of smoking.*—There were markedly fewer pipe smokers and/or cigar smokers among the patients with lung cancer than in the control group.

Inhaling of smoke as well as smoking of cigarettes was much more frequent among patients with lung cancer than in the control group.

The statistical significance of the differences in the consumption of tobacco between the patients having lung cancer and the control group are given in Table II.

*Histological subgroups.*—Patients with squamous-cell carcinoma, who comprised over half of the patients discussed, present the highest consumption of tobacco. All without exception smoked tobacco and almost all of them were " heavy " smokers (i.e. smoking index over 300), smoked cigarettes, and inhaled smoke.

Similar, yet a little lower, numbers characterize the second subgroup—"undifferentiated carcinoma or histological type not determined".

Two remaining subgroups are small and it is difficult to draw conclusions in this case. It seems that they smoke less than the patients with squamous-cell carcinoma and among them pipe smokers and/or cigar smokers are met more frequently. On the other hand they smoke more than the examined individuals in the control group.

*Other data.*—17·6 per cent of men with lung cancer had had pneumonia as had 13·1 per cent in the control group (the difference statistically is of no significance). Other diseases of lungs were rarely mentioned by the individuals in both groups.

Among the patients suffering from the lung cancer the division of blood groups was the following (for comparison the division of blood groups in Poland are given in brackets (Sabliński, 1959): A—41·7 per cent (37·1 per cent), B—19·7 per cent (18·5 per cent), AB—6·3 per cent (7·6 per cent), O—32·3 per cent (36·7 per cent). Thus no connection was found between lung cancer and some blood groups.

B. *Women* (Table III)

The small number of cases makes detailed analysis impossible and only allows a few general conclusions to be reached.

The numerical proportion of men to women for the total number of patients suffering from lung cancer amounted to 12·4 : 1, for adenocarcinoma only 1·8 : 1 and for the total of the remaining histological types 24·0 : 1.

The percentage of smokers, average index, and average intensity of smoking were higher among women suffering from lung cancer than in the control group.

There is almost no difference in women patients with adenocarcinoma in reference to smoking and the control group, but in all the remaining histological subgroups the consumption of tobacco is higher. The only woman with squamous-cell carcinoma smoked and had the index of smoking above 300 (such index was met in 1·2 per cent of women in the control group).

### *Discussion*

A. *Connection between smoking and lung cancer*

Patients of both sexes suffering from lung cancer smoke markedly more than the " general population " represented by the control group. Is it possible, however, to compare these two groups? Do they represent the same population?

As can be seen from Table I, the differences in the social background of the two groups are not great. Average age, the percentage of the inhabitants of towns and the percentage of the inhabitants of Upper Silesia are very similar. The differences in the professional structure are greater—but they could not serve for the explanation of the differences noted in tobacco consumption. In none of the subgroups of professions such a high index or percentage of smokers was found as among individuals suffering from lung cancer (average index of smoking varied from 364·4 among metal workers to 512·9 among railroad workers, and the percentage of smokers from 70·3 among the office workers up to 89·1 among the colliers—thus in the control group in not one of the subgroups of professions were the values as great as among the lung cancer patients).

It may be then accepted that, as ascertained in our material, accurate statistically highly significant connection between tobacco smoking and lung cancer is not an accidental occurrence. Whether it is a causal connection—statistical and epidemiological studies cannot give a decisive answer. It can only be stated that our studies are in conformity with the view that smoking plays a most important role in the genesis of lung cancer.

The connection between smoking and lung cancer is quite distinct for patients of both sexes with squamous-cell carcinoma and with cancer included in the subgroup as "undifferentiated carcinoma and the type histologically not determined "; it is, however, less distinct for microcellular carcinoma and adenocarcinoma. In reference to the latter, conclusions cannot be drawn upon ascertaining larger consumption of tobacco among men—due to the small number of cases and due to the low tobacco consumption among women with adenocarcinoma.

Our material is not sufficient to determine whether lung cancer possesses any connection with pipe and/or cigar smoking; if there were such a connection it would be markedly smaller than in reference to cigarette smoking.

B. *The chances of morbidity and the percentage of cases connected with smoking*

Relative chances of morbidity in reference to lung cancer which the smoker had in comparison with the non-smokers may be calculated for our material by employing a formula given by Cornfield (1951):

$$A = \frac{p_1}{p_2} \cdot \frac{(1 - p_2)}{(1 - p_1)}$$

where $A$ = relative amount by which the prevalence of lung cancer is augmented by the attribute of smoking.

$p_1$ = proportion of smokers among individuals examined with lung cancer.

$p_2$ = proportion of smokers among individuals examined in the control group.

Using this formula we find that a smoker possessed about 10-fold greater chances of becoming afflicted by lung cancer than a non-smoker.

The proportion of cases connected with smoking will then be:

$$y = \frac{A - 1}{A} \times p_1 = 88{\cdot}2 \text{ per cent.}$$

And thus in almost 90 per cent of male individuals we found a connection between smoking and lung cancer.

The same problem may be discussed in another way. Let us calculate how many men with lung cancer we would expect to meet if smoking among the entire male population were the same as among women and if there were a close correlation between the appearance of lung cancer (except adenocarcinoma) and high tobacco consumption represented by a higher index than 300. Such an index appeared in 49·1 per cent of the total number of examined men and in 1·2 per cent of the total number of women in the control group—proportion 41 : 1. And therefore a decrease in the frequency of appearance of a higher than 300 index by 41 times would also bring about a decrease in the number of men afflicted by lung cancer. We would then have not 240 but only 6 men suffering from lung cancer of a type other than adenocarcinoma and therefore a similar number as in women (10 cases after subtracting cases of adenocarcinoma). Total elimination of smoking as a carcinogenic factor would also decrease a little the number of 26 cases of lung cancer in men and 21 cases in women. It would result then that smoking played (with the above foundation) the main role in over 90 per cent of cases of lung cancer among men ($>$234/260) and in over 80 per cent of the total number of cases ($>$234/281). The numbers obtained by both methods are similar.

### *Conclusions*

Our observations prove that in Poland there is also a distinct connection between cigarette smoking and the appearance of lung cancer. The connection is observed in men as well as in women—most distinctly for squamous-cell carcinoma. Contrary to the findings of other authors the connection of smoking with microcellular carcinoma has been less distinct in Poland than with adenocarcinoma; both these subgroups were, however, too small to draw far-reaching conclusions.

The markedly more frequent appearance of lung cancer among men can be easily explained when accepting that smoking is frequently the cause of cases presenting this neoplasm.

The period from the moment of starting smoking to the appearance of symptoms of lung cancer was usually longer than 25 years (on the average it amounted to 37·1 years). Therefore a conclusion can be reached that in studying dependence between smoking habits and morbidity due to lung cancer in the examined population one should take into consideration consumption of tobacco during the last 30–40 years and not the present consumption.

It is evaluated that men smokers possessed about 10-fold greater chances of becoming afflicted by lung cancer than the non-smokers and that smoking has been connected with about 80 per cent of the total number of lung cancer cases.

### *Summary*

Results of a retrospective study conducted in Poland on tobacco smoking and lung cancer are presented. This study shows a very distinct correlation between cigarette smoking and lung cancer.

It is considered that men smokers possessed about 10-fold greater chances of becoming afflicted with lung cancer than non-smokers and that smoking was connected with about 80 per cent of the total number of lung cancer cases.

The long period from the moment of starting smoking to the appearance of symptoms of cancer points to the necessity of taking into consideration tobacco consumption during the last 30–40 years during the study of correlation between smoking habits and morbidity due to lung cancer.

The index of smoking (intensity × duration) seems to be a better criterion of classification of smokers than intensity of smoking.

### REFERENCES

Cornfield, J.—(1951) *J. nat. Cancer Inst.*, **11**, 1269.
Sabliński, J.—(1959) *Pol. med. Wkly.*, **13**, 63.
Staszewski, J.—(1958) *Nowotwory*, **8**, 51.

### PART III.—CANCER OF THE "TOBACCO TRACT" (EXCLUDING LUNG CANCER) IN MEN

During the years 1957–58 data have been collected concerning tobacco consumption, occupation, and places of residence of 394 men suffering from carcinoma and pre-cancerous conditions of the lip, 83 men with carcinoma and pre-cancerous conditions of the oral cavity, 19 with carcinoma of the tonsils, and 207 with carcinoma of the larynx.

A group of 912 men over 20 years old examined in the first half of 1957, whose reason for calling at the Institute of Oncology was most probably not connected with smoking, serves as a control group (group " C "). This group is described in general outlines in the first part of this study which discusses tobacco consumption in Poland.

Additional comparison group (group " D ") for patients with carcinoma of lip will be represented by 200 farmers afflicted by carcinoma of skin.

The manner of collecting data both for the group of examined individuals as well as in both control groups and the definitions " smokers ", " heavy smokers ", " index of smoking ", " the inhabitants of towns ", etc. are as described in Part II of this study on lung cancer.

### *Results and Conclusions*

In Table I some general data are presented in reference to patients afflicted by cancer of various sites, and in Tables II and III data on their smoking habits. We shall discuss cancer of each site in turn.

TABLE I.—*General Characteristics of Men with Cancer of the " Tobacco Tract " and of the Control Group*

| | Carcinoma and precancerous conditions of lip | Oral cavity: Carcinoma | Oral cavity: Pre-cancerous conditions | Oral cavity: Total | Carcinoma of the tonsils | Carcinoma of larynx | Control group |
|---|---|---|---|---|---|---|---|
| Number of individuals examined | 394 | 58 | 25 | 83 | 19 | 207 | 912 |
| Average age | 55·8 | 57·9 | 53·0 | 56·4 | 62·5 | 56·4 | 53·4 |
| Number and % of inhabitants of towns | 114 (28·9) | 38 (65·5) | 15 (60·0) | 53 (63·9) | 15 (78·9) | 138 (66·7) | 568 (62·3) |
| Number and % of inhabitants of Upper Silesia | 217 (55·1) | 42 (72·4) | 17 (68·0) | 59 (71·1) | 13 (68·4) | 149 (72·0) | 641 (70·3) |
| Number and % of office workers | 16 (4·1) | 11 (19·0) | 5 (20·0) | 16 (19·3) | 6 (31·6) | 26 (12·6) | 111 (12·2) |
| Number and % of farmers | 126 (32·0) | 8 (13·8) | 2 (8·0) | 10 (12·0) | 1 (5·3) | 23 (11·1) | 126 (13·8) |

1. *Cancer of lip*

In 387 patients changes concerned the lower lip and in 7 the upper lip. Histopathological diagnosis of squamous-cell carcinoma was positive in 306 patients, uncertain in 41 patients (probably carcinoma, suspicions of carcinoma), and in 47 patients only precancerous conditions were found (hyperkeratosis et proliferatio epithelii, paratypia epithelii, papilloma, leukoplakia). The patients in question were divided into 2 subgroups: farmers and non-farmers—since cancer of lip is particularly frequent in farmers, which suggests the pertinence of analysing them separately.

The data concerning the tobacco consumption in each of these subgroups is presented in Table II. Before discussing it, however, a few words will be devoted to the influence of atmospheric factors on the appearance of cancer of lip.

TABLE II.—*Smoking Habits of Men Suffering from Carcinoma and Precancerous Conditions of Lip and of the Control Group*

* For this column significance of difference was not computed.

In the group of non-farmers the percentage of individuals exposed because of their occupation to the action of atmospheric factors was markedly higher than in the control group " C ". There were in this group for instance more workers employed in the building industry (16·4 per cent in comparison with 9·0 per cent in the control group after subtracting the number of farmers), and less office workers (5·9 per cent in comparison with 14·1 per cent in the control group) or inhabitants of towns. This type of connection with the exposure to atmospheric factors was not found in the other cancers discussed later on. It is worth mentioning that among the patients with cancer and precancerous conditions of lip, farmers constitute almost one-third of the total number of patients (126 out of 394), while in the control group only about one-seventh (126 out of 912). The farmers are really particularly exposed to the action of atmospheric factors. It can be supposed then that these factors (probably the ultraviolet rays of the sun spectrum) play a large role in the appearance of precancerous conditions and cancer of lip.

Let us now take into account the tobacco consumption among the patients mentioned above.

The percentage of smokers, average intensity of smoking, average index of smoking and the percentage of heavy smokers (i.e. with the index of smoking above 300) were markedly higher among patients with cancer and precancerous conditions of lip than in the control group " C ". This is observed in all the subgroups of Table II (only in two small subgroups the percentage of heavy smokers was a bit lower than in the control group). The differences compared with the control group were larger for non-farmers (statistically significant) than for farmers for whom these differences were statistically of no importance or on the boundary of significance.

Let us compare now the farmers suffering from cancer and precancerous conditions of lip not with the control group " C " but with the additional group for comparison (" D "): 200 farmers with cancer of skin, not a large group really, but more corresponding in reference to the mode of life, conditions of work, etc., and thus representing better the consumption of tobacco among the farmers. Such a comparison brings into prominence higher tobacco consumption among farmers with cancer and precancerous conditions of lip—e.g. the percentage of smokers among them is distinctly higher than in the additional group (" D ") for comparison (the difference here exceeds 3·7 times the error of difference).

*The manner of smoking.*—Differences in reference to percentage of smokers using only cigarettes, percentage of smokers using only pipe and/or cigars, and the percentage of smokers inhaling smoke were statistically not significant; however among patients with cancer and precancerous conditions of lip there were a few more cigarette smokers and smokers inhaling smoke than in the control group. This is contrary to the results obtained by other investigators who have stated that the connection between cancer of lip and smoking is particularly distinct in smokers using a pipe and/or cigars. The reason for this disagreement is not clear. It may be caused for instance by a small number of pipe and cigar smokers among our patients—studies carried on larger material could perhaps explain that.

*Chances of morbidity and the percentage of cases connected with smoking* will be calculated similarly as for lung cancer in Part II of this study.

Relative chances of cancer and precancerous conditions of lip which a smoker has in comparison with a non-smoker amount to, after Cornfield's (1951) formula,

$$A = \frac{0{\cdot}934}{0{\cdot}827} \cdot \frac{(1 - 0{\cdot}827)}{(1 - 0{\cdot}934)} = 2{\cdot}96.$$

The percentage of cases connected with smoking amounted to:

$$y = 61{\cdot}8 \text{ per cent.}$$

Analogic computations for the subgroups of farmers when compared with the additional comparison group " D " give:

$$A = \frac{0{\cdot}905}{0{\cdot}755} \cdot \frac{(1 - 0{\cdot}755)}{(1 - 0{\cdot}905)} = 3{\cdot}09$$

$$y = 61{\cdot}2 \text{ per cent.}$$

Concluding, among our patients we find a distinct connection between the appearance of carcinoma and precancerous conditions of lip, and tobacco smoking and exposure to the action of atmospheric factors. Pipe and/or cigar smoking was not found to be more connected with the discussed diseases than smoking of cigarettes. The smoker had 3 times greater chance of becoming afflicted than the non-smoker. About 60 per cent of cases were connected with smoking.

2. *Cancer of the oral cavity*

In 58 patients the diagnosis of squamous-cell carcinoma was proved by histopathological examination and in 25 patients there were found only precancerous changes (leukoplakia, suspicious epithelial proliferation, papilloma). Differences in the smoking habits of both of these small subgroups were not great (Table III).

The percentage of smokers, average intensity of smoking, average index of smoking and the percentage of heavy smokers were significantly higher among patients suffering from cancer and precancerous conditions of the oral cavity than in the control group. But the differences in the manner of smoking were not significant from the statistical point of view. The percentage of those smoking only pipe and/or cigars and not inhaling smoke was however a little higher among the discussed patients. This is in agreement with the statement by other investigators that there is a more distinct connection between cancer of the oral cavity and smoking pipe and/or cigars than with cigarette smoking.

The relative chances of a smoker to become afflicted by cancer and precancerous conditions of the oral cavity amounted to:

$$A = \frac{0{\cdot}988}{0{\cdot}827} \cdot \frac{(1 - 0{\cdot}827)}{(1 - 0{\cdot}988)} = 17{\cdot}2.$$

This number may be considered as only a near estimate because with the small number of non-smokers it is very dependent on chance (a slight change in the percentage of smokers among patients markedly changes the number in the denominator). The percentage of cases connected with smoking amounted to:

$$y = 93{\cdot}1 \text{ per cent.}$$

Concluding, among the discussed patients there was a distinct connection between smoking, and cancer and precancerous conditions of the oral cavity.

TABLE III.—*Smoking Habits of Men with Cancer of the Oral Cavity, Tonsils and Larynx and of the Control Group*

| | Oral cavity | | | | | |
|---|---|---|---|---|---|---|
| | Carcinoma* | Precancerous conditions* | Total | Carcinoma of the tonsils* | Carcinoma of the larynx | Control group |
| Number of individuals examined | 58 | 25 | 83 | 19 | 207 | 912 |
| Number and % of smokers | 57 (98·3) | 25 (100·0) | 82 (98·8)§ | 19 (100·0) | 206 (99·5)§ | 754 (82·7) |
| Average intensity of smoking | 15·1 | 14·9 | 15·0‡ | 16·4 | 15·7§ | 12·2 |
| Average index of smoking (calculated for smokers) | 544·7 | 492·4 | 535·4‡ | 600·8 | 585·3§ | 406·3 |
| Number and % of heavy smokers (with the index over 300) | 47 (82·5) | 20 (80·0) | 67 (81·7)‡ | 13 (68·4) | 183§ (88·8) | 447 (59·3) |
| Number and % of smokers smoking only cigarettes | 39 (68·4) | 22 (88·0) | 61 (74·4) | 13 (68·4) | 182‡ (88·3) | 552 (73·2) |
| Number and % of smokers smoking only pipe and/or cigars | 12 (21·1) | 2 (8·0) | 14 (17·1) | 3 (15·8) | 4* (1·9) | 101 (13·4) |
| Number and % of smokers inhaling smoke | 40 (70·2) | 22 (88·0) | 62 (75·6) | 15 (78·9) | 199§ (96·6) | 609 (80·8) |

*Note :* The calculations of significance have been taken from the study by Dr. Różanowicz (personal conmmunication).

* For this column significance of difference was not computed.

† The difference with the control group is significant—exceeding two to three times the standard error of difference calculated in the equation

$$S.E. = \sqrt{\frac{p_1 q_1}{n_1} + \frac{p_2 q_2}{n_2}}$$

‡ The difference with the control group is distinctly significant—exceeding three to six times the standard error of difference.

§ The difference with the control group is exceedingly significant—exceeding at least six times the standard error of difference.

The smoker had about 17 times greater chance of becoming afflicted than the non-smoker. About 90 per cent of cases were connected with smoking.

### 3. *Carcinoma of tonsils*

In this small group in all cases the diagnosis of squamous-cell carcinoma was confirmed by histopathological findings.

All the patients were smokers. Average intensity and average index of smoking and the percentage of heavy smokers were markedly higher than in the control group, but the manner of smoking did not differ in both groups. Due to the small number of patients with carcinoma of tonsils statistical appraisal of these correlations is not possible.

### 4. *Carcinoma of larynx*

In all the patients the diagnosis of squamous-cell carcinoma was confirmed by histopathological findings. Extrinsic larynx cancer was also included here. There were only slight differences in the smoking habits of the patients with cancer of the larynx of various localizations.

The percentage of smokers, average intensity of smoking, average index of smoking and percentage of heavy smokers were markedly higher among these patients than in the control group (the differences being statistically extremely significant).

The percentage of smokers using only cigarettes as well as the percentage of those inhaling smoke was among the patients with cancer of larynx markedly higher than in the control group; these percentages were also higher than in patients with cancer of lip, cancer of the oral cavity, or tonsils—and very similar to the percentages observed in lung cancer (see Part II of this study).

The relative chances of morbidity for smokers amounted to:

$$A = \frac{0 \cdot 995}{0 \cdot 827} \cdot \frac{(1 - 0 \cdot 827)}{(1 - 0 \cdot 995)} = 41 \cdot 6.$$

The number obtained above is only of orientation significance because of the very small number of non-smokers—it would greatly change if for instance instead of one there were two non-smokers. The obtained number however proves that the smoker had many more chances of becoming afflicted by cancer of larynx than the non-smoker.

The percentage of cases connected with smoking amounted to:

$$y = 97 \cdot 1 \text{ per cent.}$$

Concluding, among the discussed patients cancer of larynx is very distinctly connected with smoking and particularly with cigarette smoking and inhalation of smoke. The smoker had about 40 times greater chance of becoming afflicted by cancer of larynx than the non-smoker. Over 90 per cent of cases afflicted by the said tumour were connected with smoking.

5. *Women*

Among 31 examined women, not included in the Tables (13 with carcinoma and precancerous conditions of lip, 3 with carcinoma of the oral cavity, 2 of the tonsils, and 13 with carcinoma of larynx) 7 were smokers (2 with carcinoma of lip, 1 carcinoma of the oral cavity, and 4 carcinoma of larynx) in whom the average index of smoking amounted to 307·9. These numbers are not sufficient to draw far reaching conclusions. They show however higher tobacco consumption among women with the discussed tumours than in the control group of 1813 women (discussed in Part I of this study, where 8·4 per cent were smokers and the average index of smoking among the smokers amounted to 142·6).

### *Discussion*

The patients with the " tobacco tract " cancer smoked markedly more than the general population represented by the control group. Is the observed connection accidental, caused by not appropriate selection of the control group? In other words—are the two groups comparable? Do they represent the same population?

Judging by Table I, the differences in the social background between patients with cancer of larynx or of the oral cavity and the control group " C " are not great since the average age, percentage of inhabitants of towns or inhabitants of Upper Silesia as well as occupational structure of these groups are similar. The reason for the difference in structure of the group of patients afflicted by cancer of tonsils may be the small number of patients in this group. Among the patients suffering from cancer of lip, the previously discussed differences in the occupational structure and first of all a large percentage of farmers is noted. The farmers were placed in a separate group having an additional group " D " for comparison.

In none of the occupational subgroups of the control group higher percentage of smokers than 89·1, or average index of smoking higher than 512·9 were noted, and the differences in the smoking habits between the inhabitants of towns and rural areas were not great. The differences in the tobacco consumption between the control group and the patients discussed cannot therefore be explained by sampling error. The evaluation of statistical significance of these differences speaks against their accidental appearance. It should be accepted then that there exists a distinct connection between the appearance of the discussed neoplasms in Poland and tobacco smoking.

### *Summary*

Results of a retrospective study conducted in Poland on tobacco smoking and cancer of lip, oral cavity, tonsils and larynx are presented. This study showed a distinct correlation between tobacco smoking and the above-mentioned cancers among men.

The relative chances of morbidity of smokers and the percentage of cases connected with smoking are computed.

The number of women examined was small but even among them tobacco consumption was found to be higher than on the average.

The author avails himself of the opportunity to express his gratitude to the Director of the Institute of Oncology in Gliwice—Dr. Święcki, M.D.—for encouragement and valuable advice, and to Mr. Dębogórski, Master of Laws, Director of the Polish Tobacco Industry, and to his co-workers: Mr. Trojan, Doctor of Chemistry, Mr. Trzciński, Doctor of Agricultural Sciences, Mr. Compala, Master of Chemistry, Mr. Wierzba, Master of Agricultural Sciences—for their friendliness which rendered possible this study.

### REFERENCE

Cornfield, J.—(1951) *J. nat. Cancer Inst.*, 11, 1269.


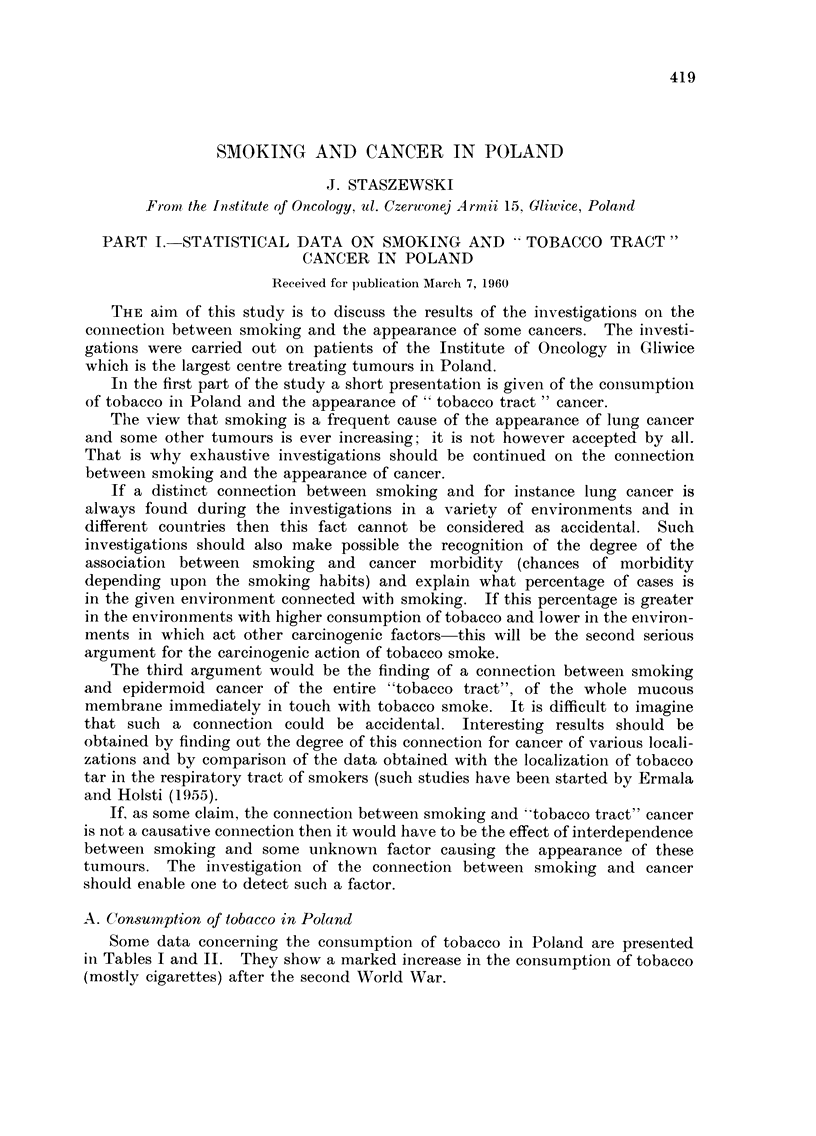

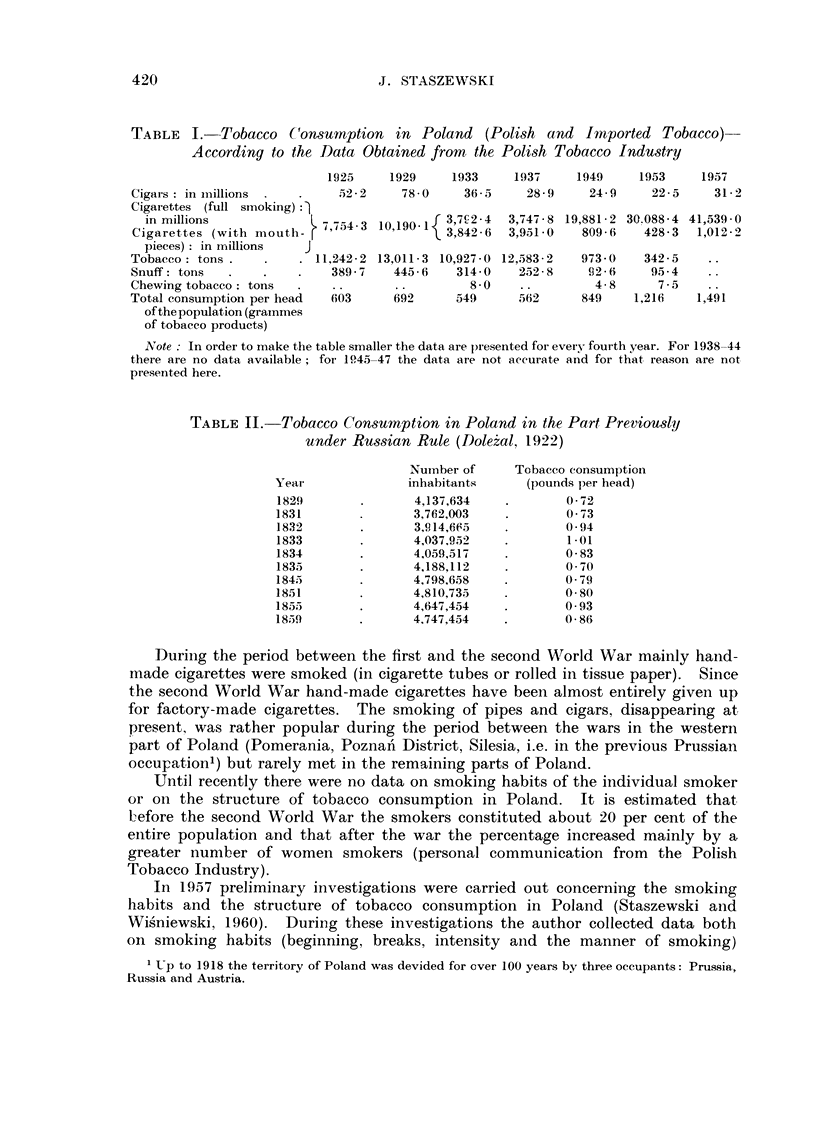

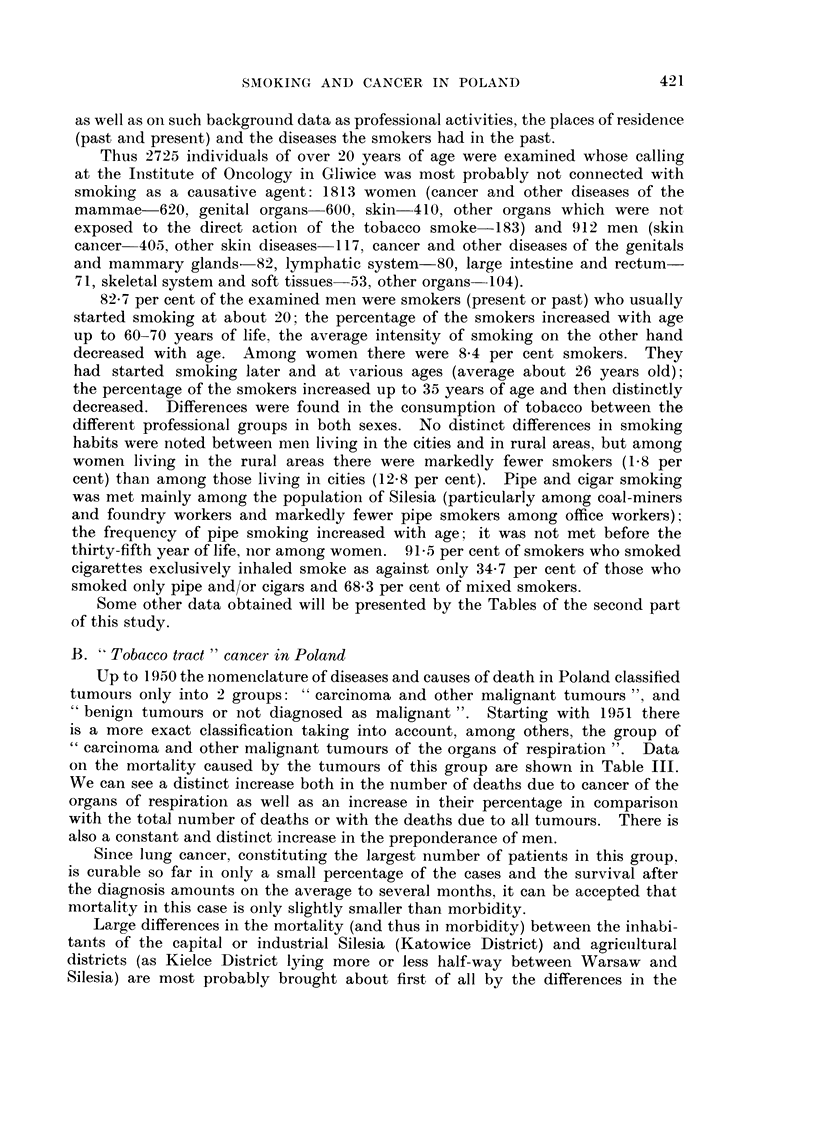

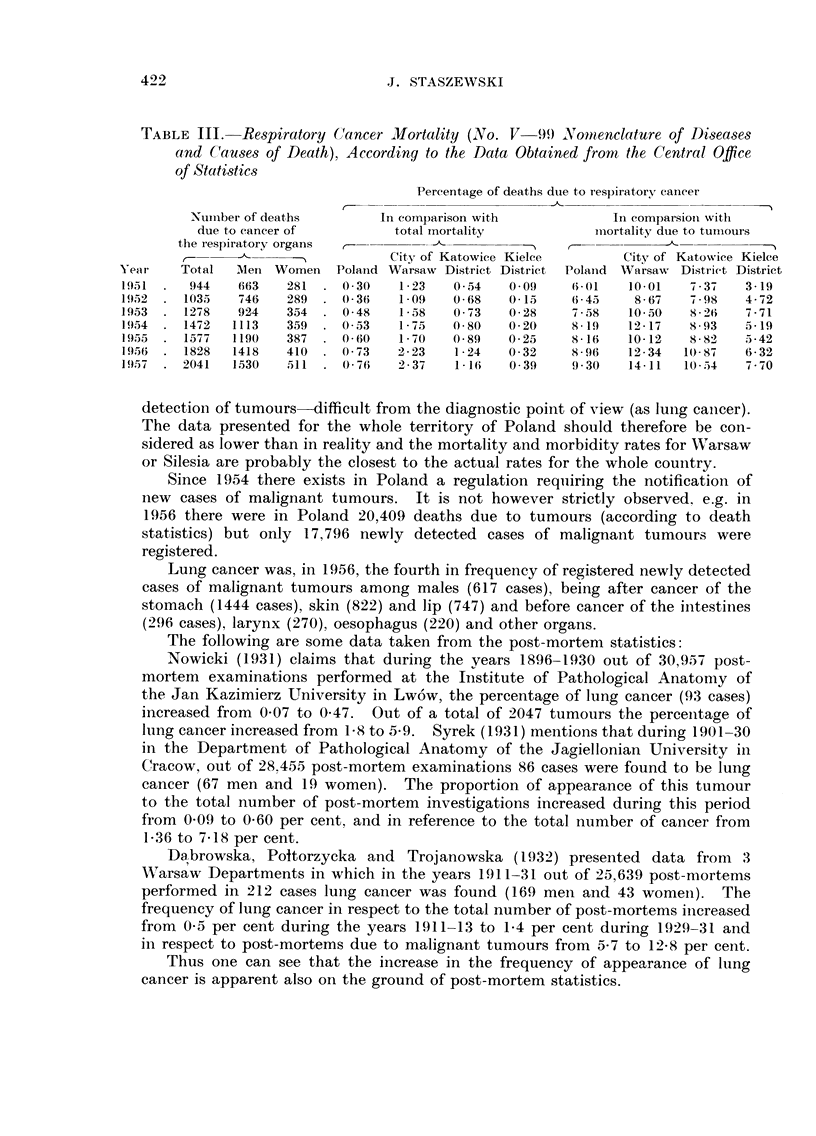

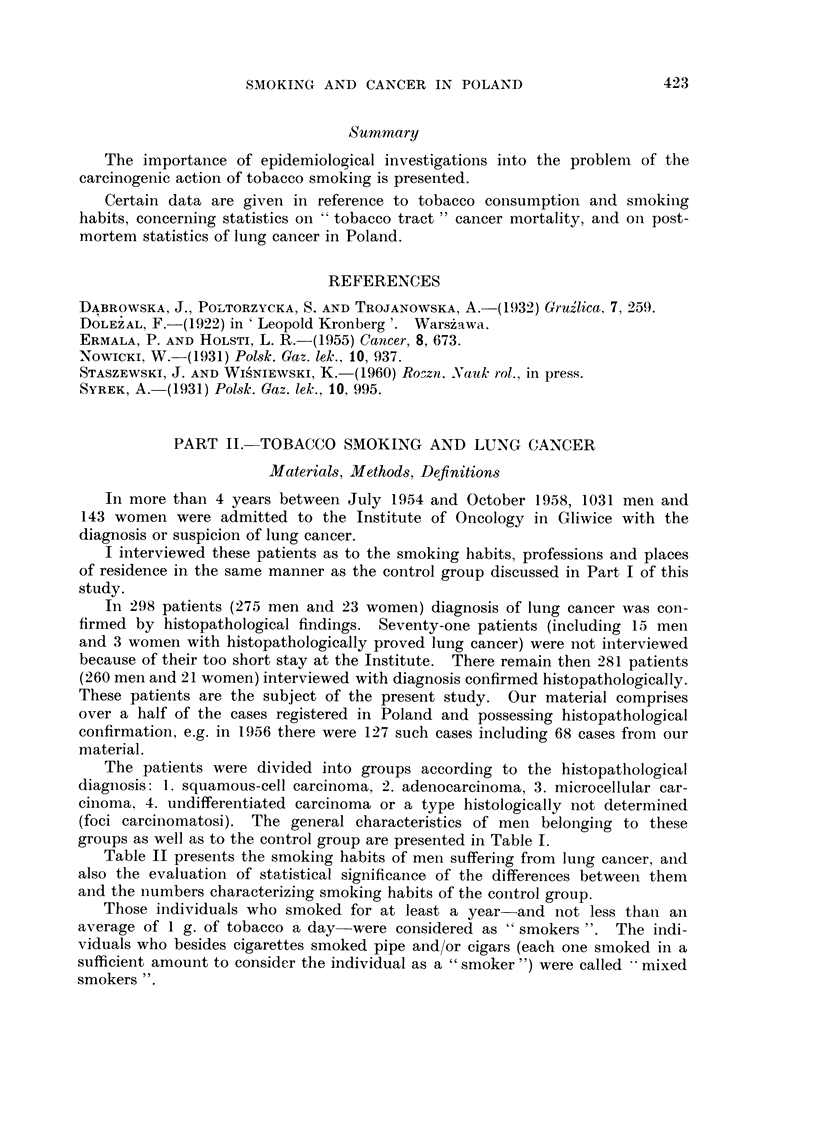

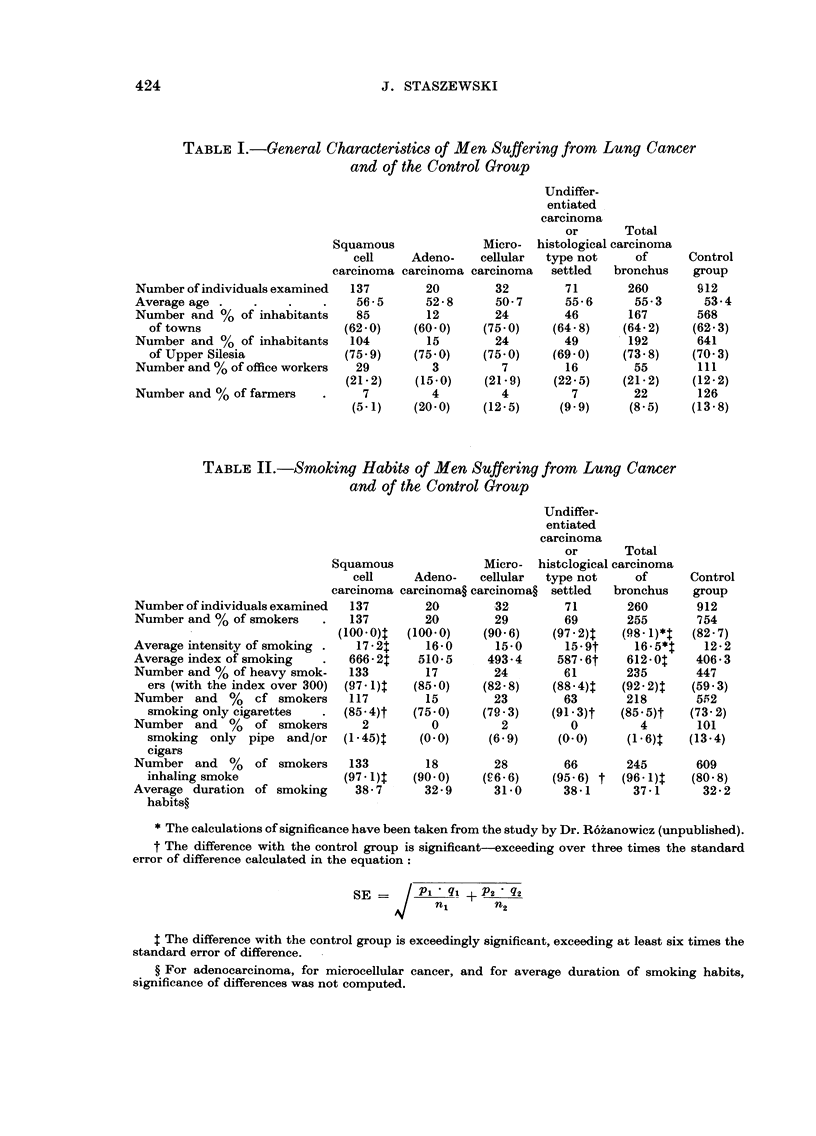

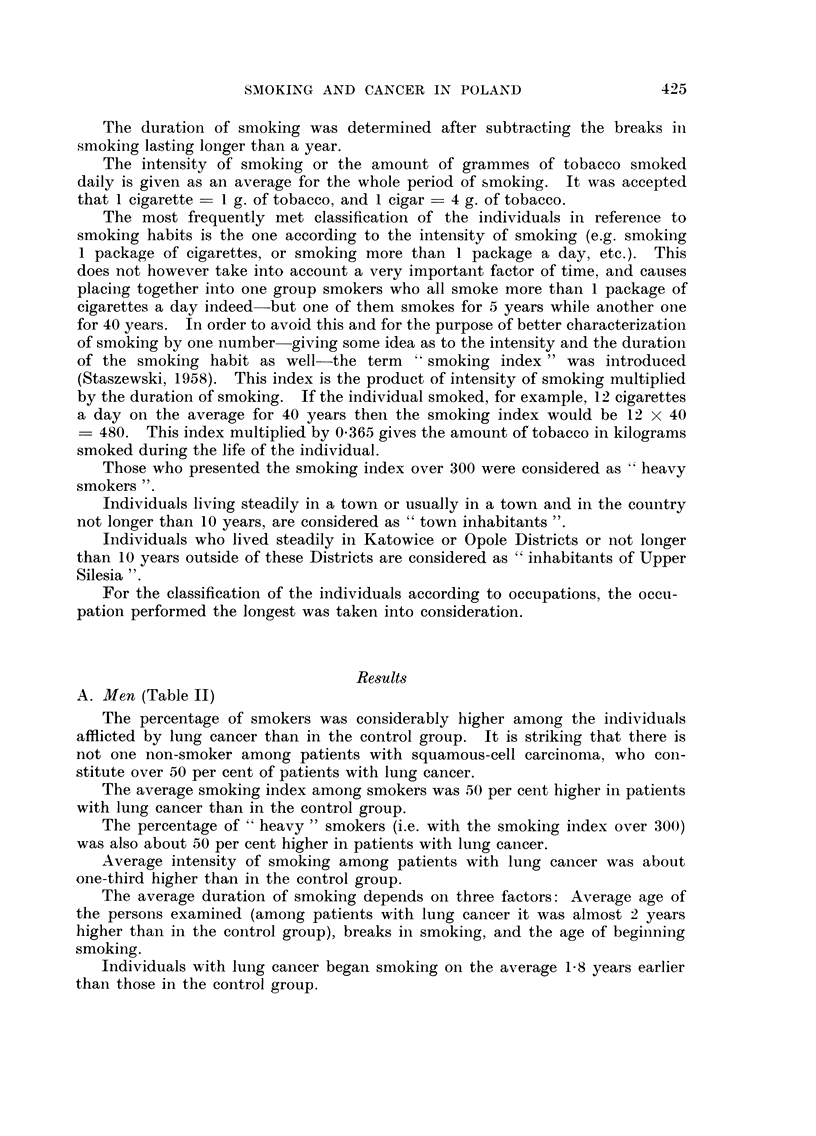

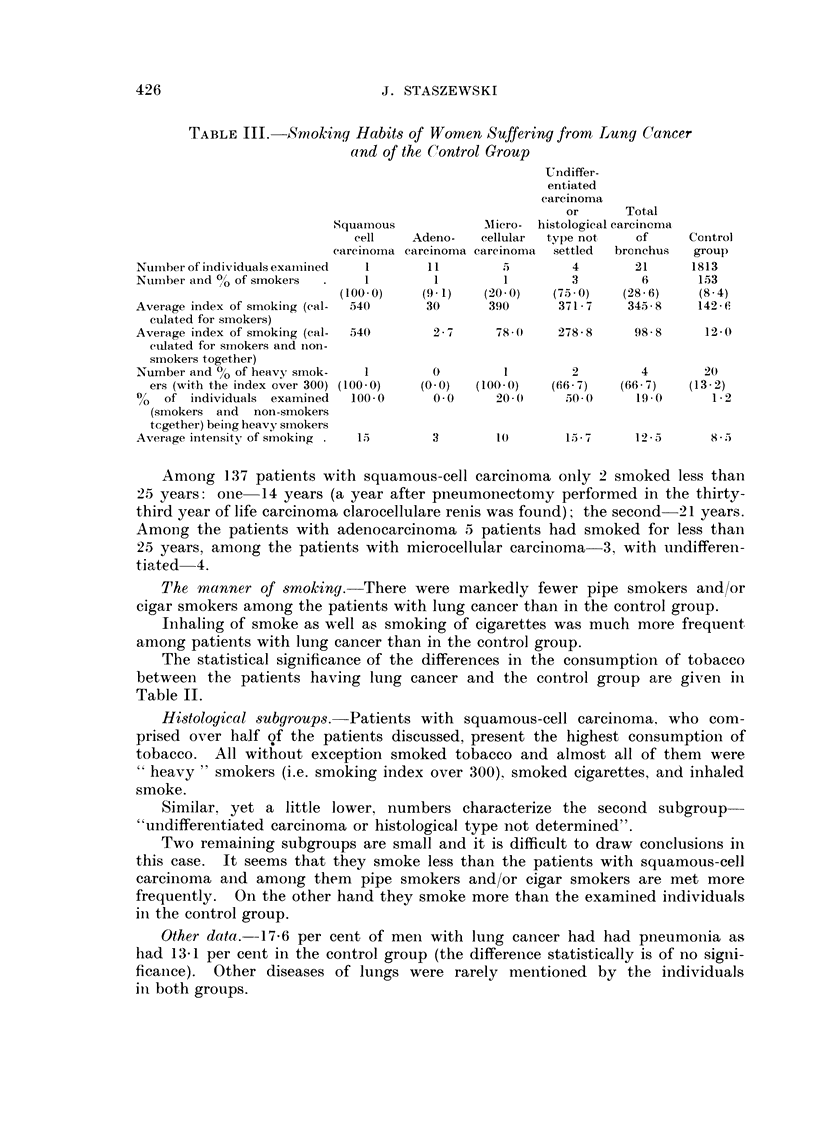

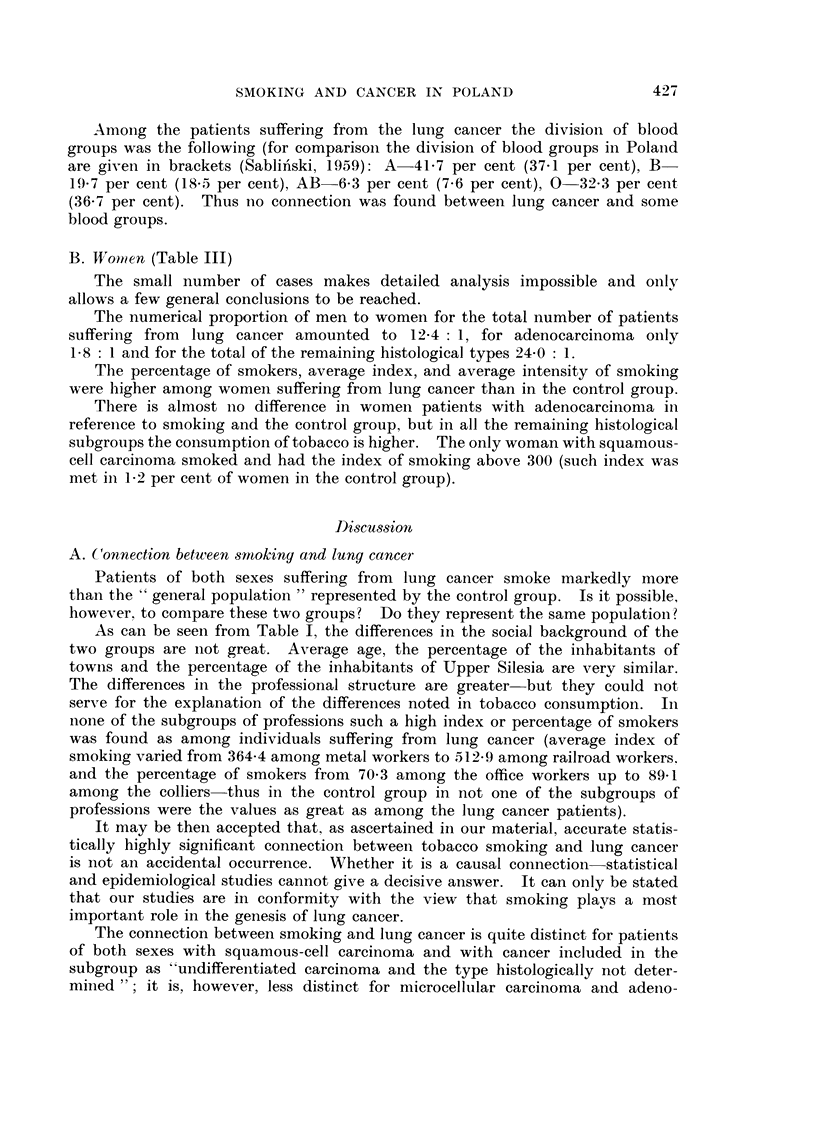

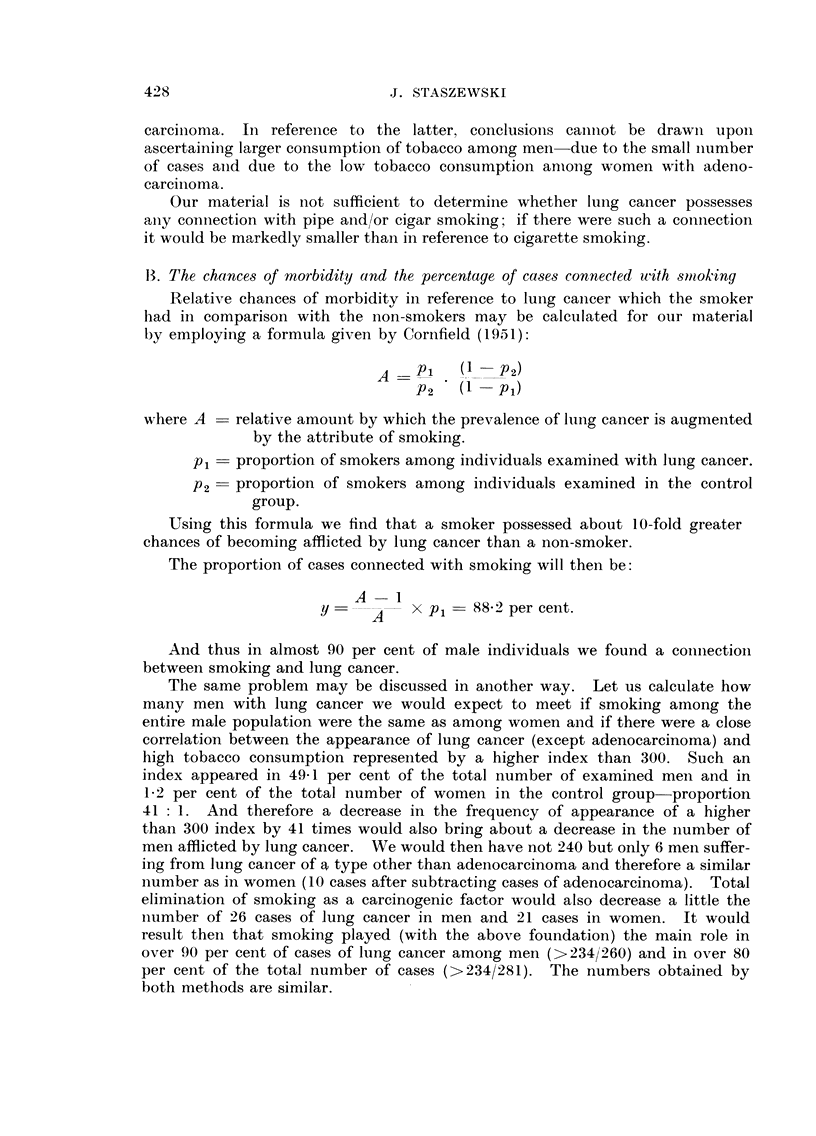

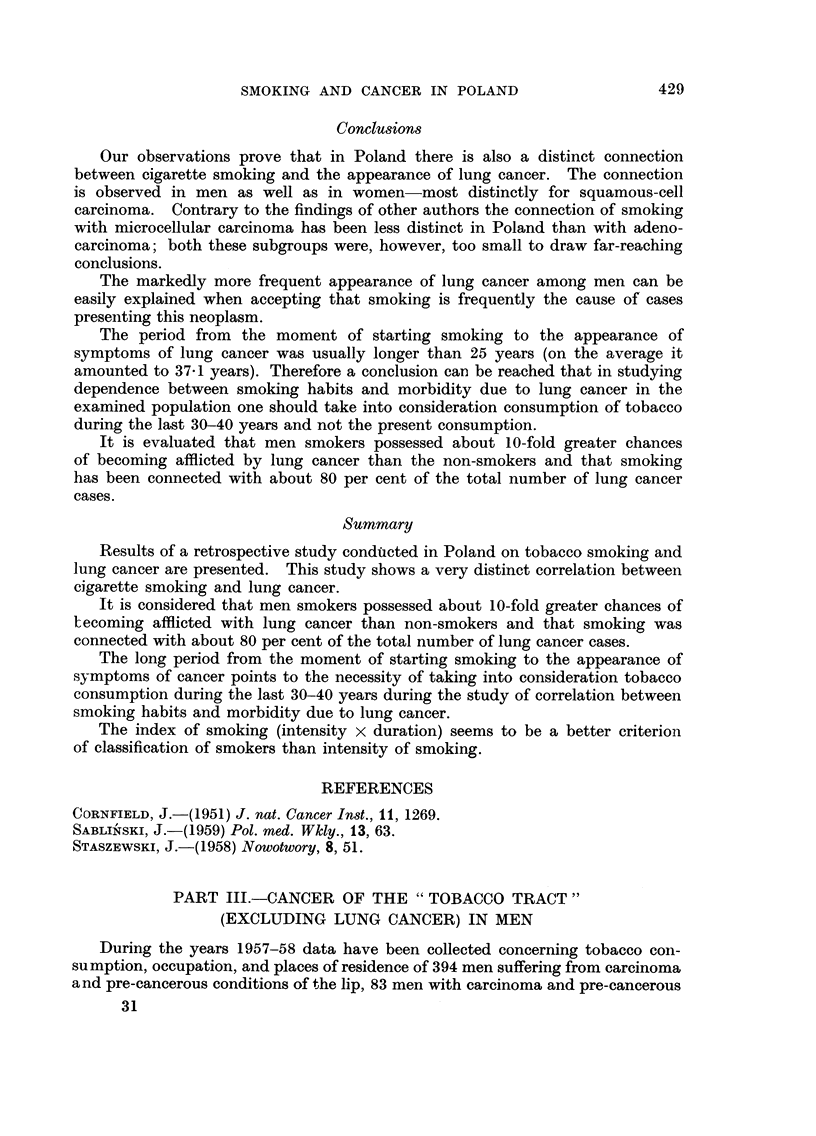

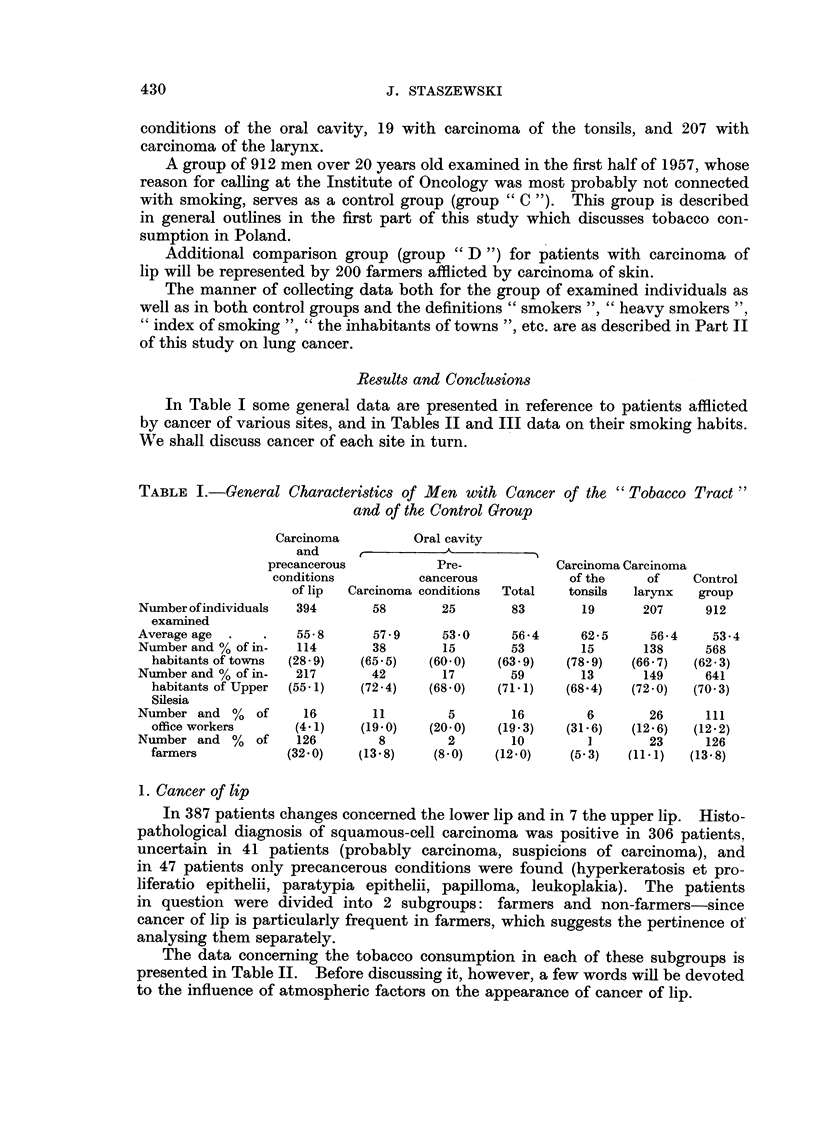

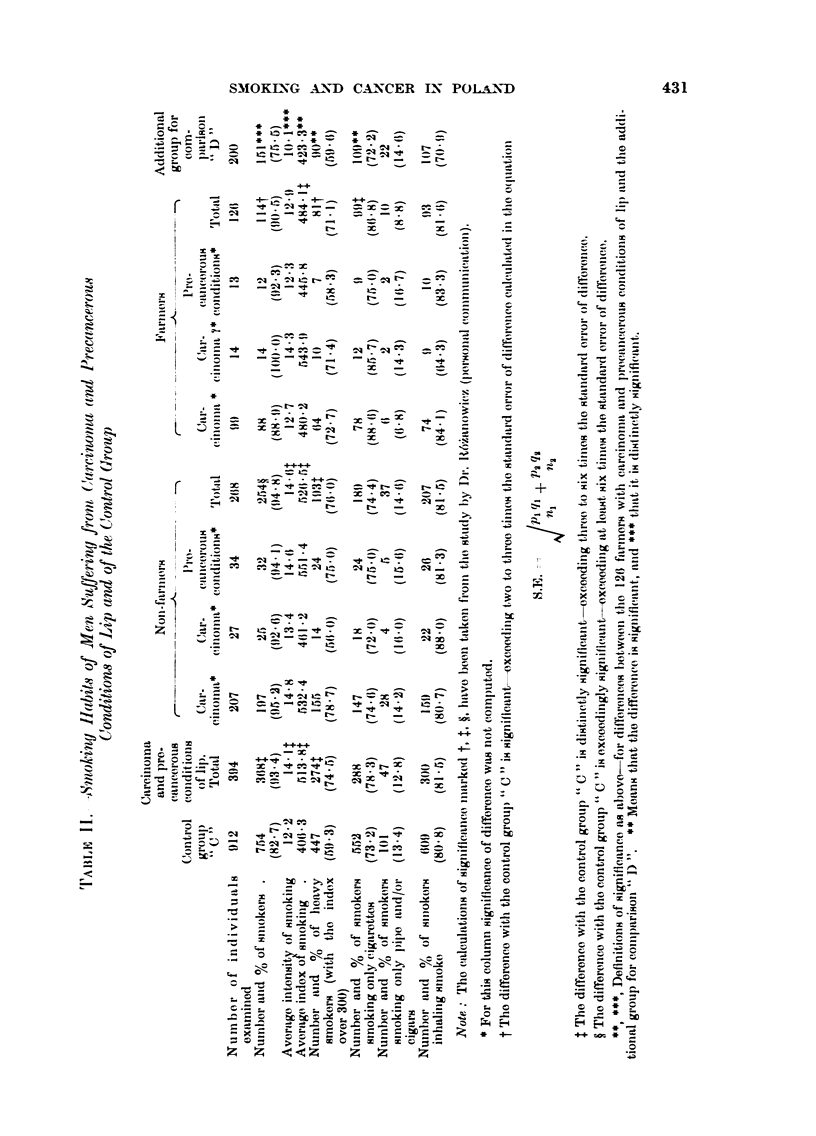

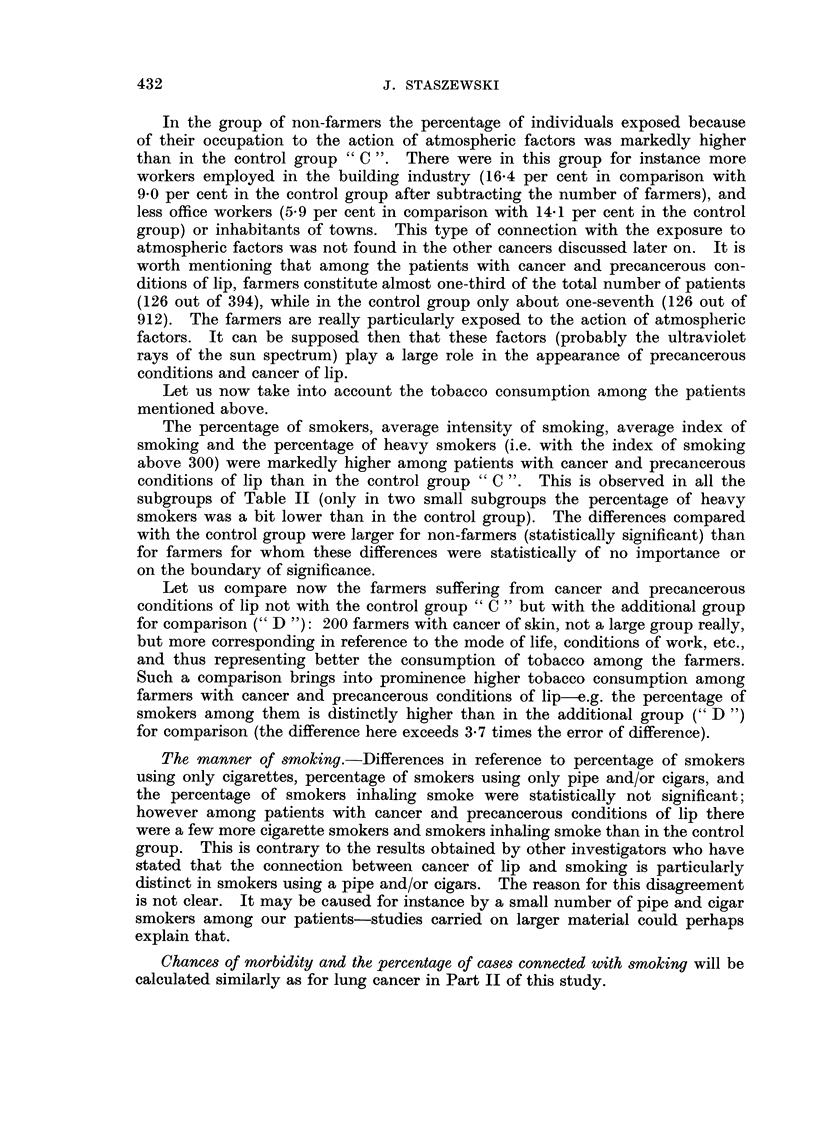

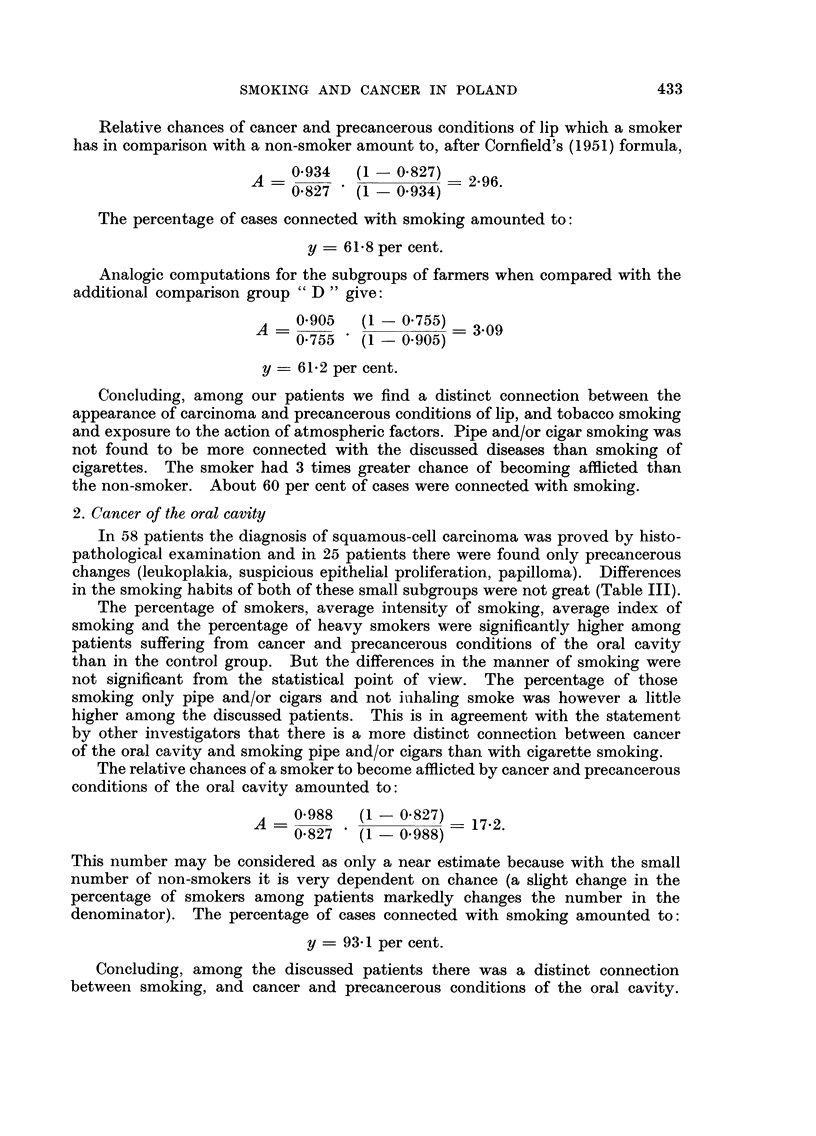

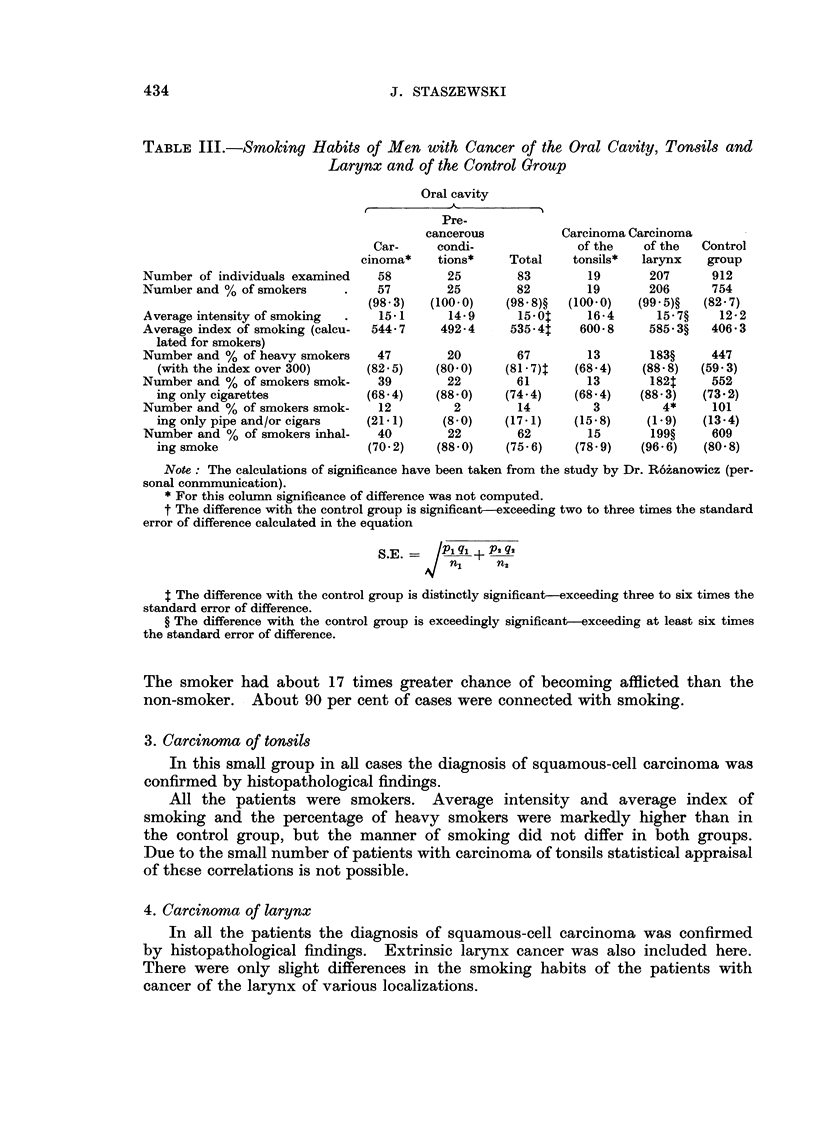

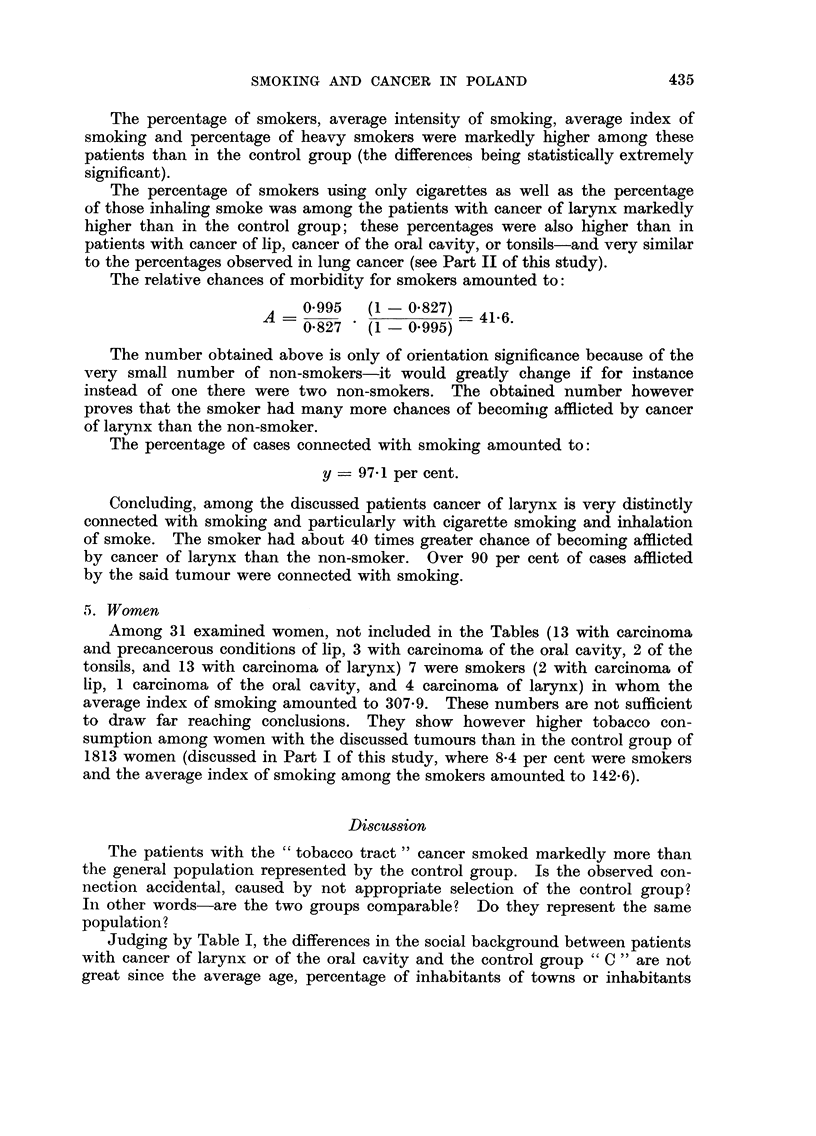

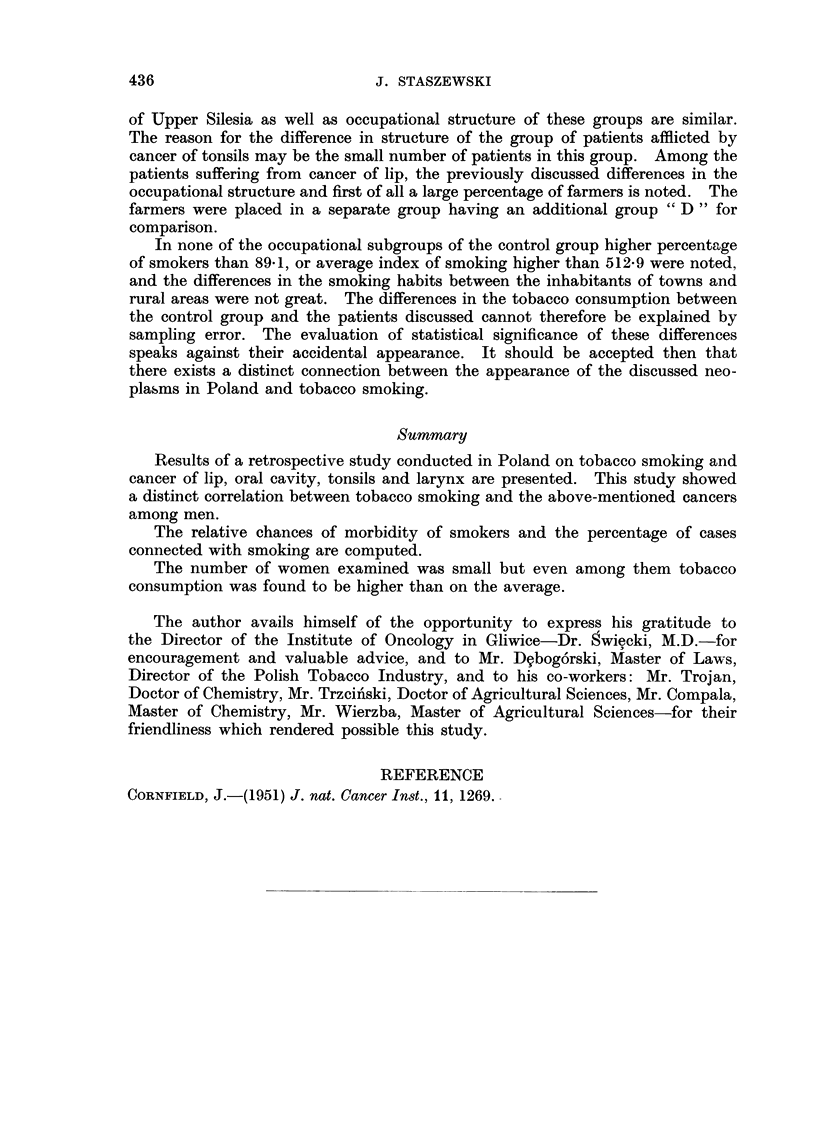


## References

[OCR_00868] CORNFIELD J. (1951). A method of estimating comparative rates from clinical data; applications to cancer of the lung, breast, and cervix.. J Natl Cancer Inst.

[OCR_00279] ERMALA P., HOLSTI L. R. (1955). Distribution and absorption of tobacco tar in the organs of the respiratory tract.. Cancer.

